# Effusion Reduction after Early Trastuzumab Deruxtecan Therapy for Unresectable HER2-Low Breast Cancer in an Older Patient: A Case Report

**DOI:** 10.70352/scrj.cr.25-0466

**Published:** 2025-12-10

**Authors:** Ayaka Akabane, Masaaki Kawai, Kazushi Suzuki, Ayaka Goto, Takayuki Tanaka, Mitsuru Futakuchi, Fuyuhiko Motoi

**Affiliations:** 1 Department of Surgery I, Yamagata University Faculty of Medicine, Yamagata, Yamagata, Japan; 2 Department of Pathology, Yamagata University Faculty of Medicine, Yamagata, Yamagata, Japan

**Keywords:** HER2-low breast cancer, trasutuzumab deruxtecan, older patient, pleural effusion

## Abstract

**INTRODUCTION:**

In recent years, HER2-low breast cancer has emerged as a distinct subtype, and the anti-HER2 antibody–drug conjugate trastuzumab deruxtecan (T-DXd) has become a promising treatment option. We report the case of an older patient with advanced HER2-low breast cancer resistant to endocrine therapy, who demonstrated a favorable clinical response to the early introduction of T-DXd.

**CASE PRESENTATION:**

A 70-year-old woman was diagnosed at the age of 64 years with stage IV right breast cancer (invasive lobular carcinoma; T1cN1M1 with pleural effusion positive for malignant cells). The patient received endocrine therapy and a CDK4/6 inhibitor; however, disease progression was observed, including increased pleural and ascitic effusion. Cytotoxic chemotherapy was discontinued owing to adverse events and impaired activities of daily living (ADL). Low HER2 expression was confirmed in ascitic cell block specimens, and T-DXd therapy was initiated. After 4 courses of T-DXd, the pleural and ascitic effusions were almost resolved, and no further paracentesis was required. The patient’s ADL improved, and she experienced clinical benefit for 15 months without any severe adverse events.

**CONCLUSIONS:**

T-DXd is potentially a safe and effective treatment option for older patients with HER2-low breast cancer and for those with impaired ADL. Further accumulation of cases and investigations of long-term outcomes are warranted.

## Abbreviations

ADL
: activities of daily living
ECOG-PS
: Eastern Cooperative Oncology Group Performance Status
ER
: estrogen receptor
HR
: hormone receptor
IHC
: immunohistochemistry
ILD
: interstitial lung disease
LI
: labeling index
PFS
: progression-free survival
PgR
: progesterone receptor
T-DXd
: trastuzumab deruxtecan

## INTRODUCTION

Subtype classification has long played a central role in guiding the treatment strategies for breast cancer. Endocrine therapy is the primary treatment for HR-positive, HER2-negative breast cancer. Recently, HER2-low breast cancer, defined as IHC 1+ or 2+ with negative *in situ* hybridization, has gained attention, with approximately 60% of previously defined HER2-negative tumors being reclassified as HER2-low.^1)^ Based on the results of the DESTINY-Breast04 trial,^2)^ T-DXd was approved in Japan on March 27, 2023, for patients with unresectable or recurrent HER2-low breast cancer following chemotherapy.^3)^

Herein, we report a case of advanced HER2-low breast cancer in which T-DXd led to a therapeutic response, accompanied by a review of the relevant literature.

## CASE PRESENTATION

**Fig. 1:**
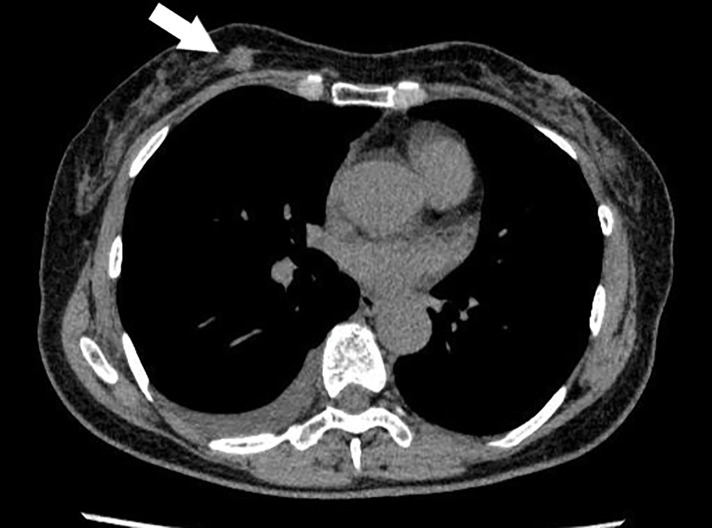
The primary tumor in the right breast (arrow) and associated right-sided pleural effusion. Diagnostic thoracoscopy subsequently confirmed pleural dissemination of invasive lobular carcinoma.

The patient was a 70-year-old postmenopausal woman (ECOG-PS: 2). Past medical history included surgery for endometrial carcinoma, osteoporosis, lumbar spinal canal stenosis, and kyphosis. At the age of 64, the patient was diagnosed with right invasive lobular carcinoma (T1cN1M1 with pleural effusion positive for malignant cells) following thoracoscopy for pleural effusion (**Fig. 1**). The initial diagnosis was stage IV luminal B disease based on pleural nodule biopsy findings (ER+, PgR+, HER2 IHC+, and Ki-67 LI 50%). Subsequently, the primary breast lesion was excised surgically for histopathological confirmation of diagnosis, rather than for therapeutic intent. Pathology revealed an invasive lobular carcinoma of the breast, luminal A subtype (ER 99%, PgR 70%, HER2 IHC 1+, and Ki-67 LI 9%).

Endocrine therapy was initiated with tamoxifen for 9 months, followed by palbociclib and fulvestrant; 53 months later, at the age of 68 years, the patient developed increasing pleural and ascitic effusions, indicating disease progression. Owing to her poor baseline ADL, intravenous chemotherapy was deemed unsuitable, and oral chemotherapy agents S-1 and capecitabine were administered. However, each treatment was discontinued after 1 cycle owing to adverse events: S-1 was discontinued due to rash, possibly allergic in nature, and capecitabine was discontinued due to diarrhea and pancytopenia. These adverse events further reduced her ADL, resulting in wheelchair dependency. Management was shifted to aromatase inhibitor therapy, while supportive care included repeated paracentesis and administration of diuretics.

**Fig. 2:**
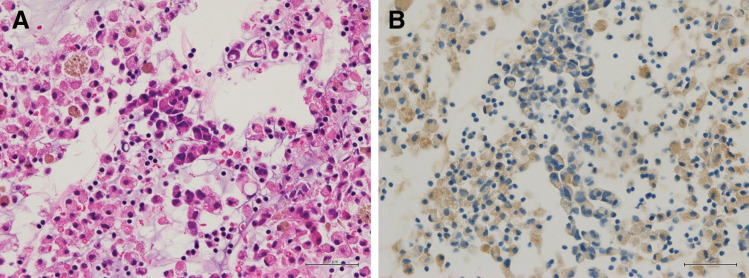
Pathological findings of ascitic fluid cell block specimens. (**A**) Hematoxylin and eosin staining (×200). (**B**) HER2 IHC showing 1+ expression (×200). IHC, immunohistochemistry

**Fig. 3:**
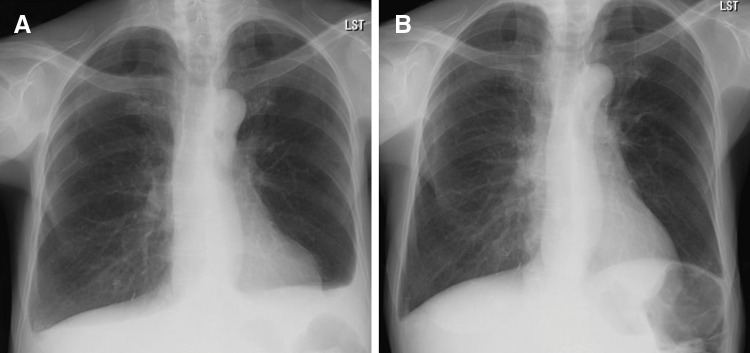
Changes in right-sided pleural effusion before and after T-DXd therapy. (**A**) Before initiation of T-DXd therapy, showing a right-sided pleural effusion. (**B**) After initiation of T-DXd therapy, showing a reduction in right-sided pleural effusion. T-DXd, trastuzumab deruxtecan

Seven months after the initiation of aromatase inhibitor therapy, when the patient was 69 years old, following the approval of T-DXd for HER2-low breast cancer, cytology of ascitic fluid confirmed HER2-low luminal B disease (ER+, PgR+, HER2 IHC 1+ by VENTANA 4B5, Ki-67 LI 20%–30%) (**Fig. 2**). Although T-DXd is usually administered to patients with ECOG PS 0–1, as an antibody–drug conjugate with relatively low systemic toxicity, it was considered feasible in this PS2 patient and was therefore selected as the next-line treatment. T-DXd was initiated, leading to resolution of the pleural and ascitic effusions after 4 courses (**Figs. 3**[Fig F4]–**5**). No further paracentesis was needed, and the patient’s ADL significantly improved. Given the presence of pleural effusion, T-DXd–related ILD could have posed a serious risk; therefore, the patient underwent close monitoring for respiratory symptoms and routine imaging. The patient maintained a good condition without notable adverse events, but 15 months after initiating T-DXd, the effusion recurred. Accordingly, T-DXd was discontinued, and palliative care was initiated. The patient transitioned to home-based care 4 months later and passed away at the age of 71 years due to breast cancer.

**Fig. 4 F4:**
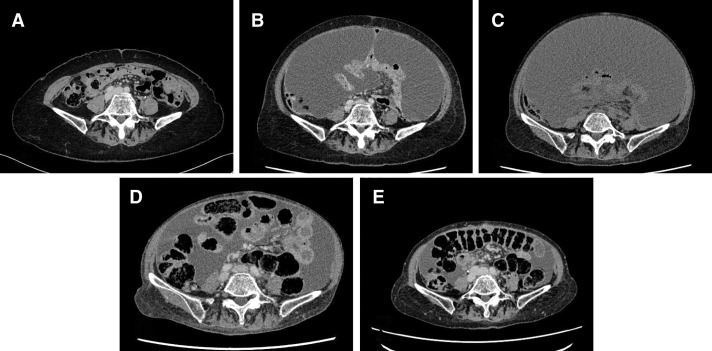
Serial axial CT images showing changes in ascites during the course of treatment. (**A**) During continued treatment with fulvestrant. (**B**) At initiation of S-1. (**C**) Prior to initiation of T-DXd. (**D**) After 4 cycles of T-DXd. (**E**) During maintenance phase of T-DXd therapy. The initiation of T-DXd therapy resulted in a reduction in ascites, which was sustained over time and contributed to an improvement in the patient’s ADL. ADL, activities of daily living; T-DXd, trastuzumab deruxtecan

**Fig. 5:**
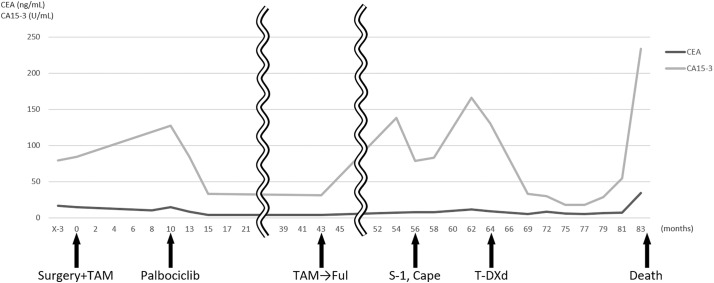
Tumor marker levels during the treatment course. After initiation of T-DXd, tumor markers were at the lowest levels observed during the entire treatment course, indicating a favorable therapeutic response. T-DXd, trastuzumab deruxtecan

## DISCUSSION

According to the 2022 Japanese Breast Cancer Clinical Practice Guidelines, the standard first-line treatment for postmenopausal HR-positive, HER2-negative metastatic breast cancer includes a non-steroidal aromatase inhibitor combined with a CDK4/6 inhibitor.^4)^ Palbociclib and abemaciclib were approved in Japan in December 2017 and November 2018, respectively. In this case, palbociclib was initiated after approval and was effective for approximately 4 years.

Disease progression with recurrent effusions eventually occurred. Owing to her general frailty and decreased physical function, intravenous chemotherapy was not tolerated, and oral chemotherapy was ineffective owing to adverse events. The approval of T-DXd by VENTANA 4B5 for HER2-low tumors in March 2023 prompted reassessment via ascitic fluid cell block, confirming HER2-low expression. T-DXd was introduced without serious adverse events and yielded rapid symptom control and improvement in ADL.

T-DXd, an antibody–drug conjugate, selectively targets HER2-low tumor cells and has relatively low systemic toxicity. Intravenous dosing avoids the challenges of oral medication adherence, making it a viable option for older patients with a poor functional status. On June 27, 2025, the current breast cancer treatment guidelines were updated to include information on biomarker testing using cell-block specimens, following the expanded approval of such testing under Japan’s national health insurance system. Although there is currently no established evidence supporting the validity of diagnosing HER2-low expression using cell-block specimens,^5)^ in this case, the treatment selected based on such an evaluation was effective, suggesting the potential validity of this approach.

In the DESTINY-Breast04 study, T-DXd showed PFS of 10.1 months and an overall survival of 23.9 months in pretreated HR-positive, HER2-low advanced breast cancer. DESTINY-Breast06 showed a PFS of 13.2 months even in patients without prior chemotherapy. Our patient achieved approximately 15 months of clinical benefit and shared clinical characteristics with the DESTINY-Breast06 cohort, supporting the appropriateness of this treatment strategy.

Although T-DXd is associated with nausea, neutropenia, liver dysfunction, and cardiotoxicity, the effects are generally manageable. However, ILD is a potentially fatal adverse event, occurring in 12.1% of patients in DESTINY-Breast04 and 11.3% in DESTINY-Breast06.^2)^ Given the underlying pleural effusion and risk of respiratory compromise in the present case, the absence of ILD was crucial for a favorable outcome. Many ILD cases have been reportedly low grade,^6)^ and with careful monitoring of respiratory symptoms, SpO_2_, and imaging, early detection and intervention can ensure continued treatment safety and efficacy.

## CONCLUSIONS

This case illustrates the efficacy of early T-DXd administration for HER2-low breast cancer in an older patient and may inform treatment decisions in similar clinical scenarios. Further case studies and outcome analyses are needed to confirm these findings.

## DECLARATIONS

### Availability of data and materials

The datasets analyzed during the current study are available from the corresponding author upon reasonable request.

## References

[1] Tarantino P, Hamilton E, Tolaney SM, et al. HER2-low breast cancer: pathological and clinical landscape. J Clin Oncol 2020; 38: 1951–62. 32330069 10.1200/JCO.19.02488

[2] Modi S, Jacot W, Yamashita T, et al. Trastuzumab deruxtecan in previously treated HER2-low advanced breast cancer. N Engl J Med 2022; 387: 9–20. 35665782 10.1056/NEJMoa2203690 PMC10561652

[3] Hattori M, Honma N, Nagai S, et al. Trastuzumab deruxtecan for human epidermal growth factor receptor 2-low advanced or metastatic breast cancer: recommendations from the Japanese Breast Cancer Society Clinical Practice Guidelines. Breast Cancer 2024; 31: 335–9. 38433181 10.1007/s12282-024-01550-0 PMC11045609

[4] Yamamoto Y, Yamauchi C, Toyama T, et al. The Japanese Breast Cancer Society clinical practice guidelines for breast cancer, 2022 edition: changes from the 2018 edition and general statements on breast cancer treatment. Breast Cancer 2024; 31: 340–6. 38570435 10.1007/s12282-024-01566-6 PMC11045566

[5] Nishimura R, Okamoto N, Satou M, et al. HER 2 immunohistochemistry for breast cancer cell blocks can be used in the same way as that used for histological specimens. Diagn Cytopathol 2016; 44: 274–9. 26800514 10.1002/dc.23433 PMC4819718

[6] Powell CA, Modi S, Iwata H, et al. Pooled analysis of drug-related interstitial lung disease and/or pneumonitis in nine trastuzumab deruxtecan monotherapy studies. ESMO Open 2022; 7: 100554. 35963179 10.1016/j.esmoop.2022.100554 PMC9434416

